# PEComa of the Upper Extremity: A Unique Case and Description of an Initial Response to Neoadjuvant Chemotherapy

**DOI:** 10.1155/2007/53056

**Published:** 2007-12-16

**Authors:** D. A. Osei, F. Alvandi, J. S. Brooks, C. M. Ogilvie

**Affiliations:** ^1^University of Pennsylvania School of Medicine, Philadelphia, Pennsylvania 19104, USA; ^2^Department of Pathology, Pennsylvania Hospital, University of Pennsylvania Health Systems, PA 19107, USA; ^3^Department of Orthopaedic Surgery, Pennsylvania Hospital, University of Pennsylvania Health Systems, PA 19106, USA

## Abstract

*Purpose*. Tumors of the perivascular epithelial cell tumor (PEComa), first described in 1992, represent a rare soft tissue neoplasm of varying malignant potential. Cases of PEComa have been previously described in a few somatic and visceral sites, most notably in the gastrointestinal tract, genitourinary tract, and one extremity case in the thigh. To date, most malignant cases of PEComa have been resistant to chemotherapy, and as such, an appropriate therapy is not known. *Case report*. Here we describe the first case of PEComa of the upper extremity. Open biopsy revealed a high-grade malignant lesion, and the patient subsequently underwent both neoadjuvant therapy with doxorubicin, ifosfamide and mensa, and radiation therapy prior to wide surgical resection. After six cycles of chemotherapy, the tumor underwent an 80% reduction in size. Subsequent neoadjuvant radiation therapy of 5000 cGy did not further reduce the size of the tumor. Following limb sparing radical resection, pathology showed 20% necrosis within a high-grade malignant lesion. Twenty one months after beginning treatment, the patient shows no sign of local recurrence, but metastatic disease was confirmed after resection of a lung nodule. *Conclusion*. Given the favorable albeit partial response seen in this patient, the course of therapy outlined here may represent a good starting point for neoadjuvant treatment in a tumor with a historically bleak prognosis. In addition, the diagnosis of PEComa must now be entertained in the differential diagnosis of upper extremity soft tissue sarcoma.

## 1. INTRODUCTION

Perivascular epitheloid cell tumor, or PEComa, is a rare soft tissue tumor characterized by cells epitheloid in morphology with clear or eosinophilic cytoplasm, and a perivascular distribution. Immunohistologically, PEComa is defined by positive staining for the melanocytic marker HMB-45 and smooth muscle actin (SMA), but negative staining for melanocytic marker S100 and variable staining for MelanA. First described in 1992 by Bonetti et al. [1], PEComas have since been reported involving the bladder, colon, falciform ligament, uterus, kidney, skull, and the thigh [2–8]. PEComa exhibits a wide variety of behavior, from benign disease treatable by excision alone, to most recently described malignant disease with a poor chemotherapeutic response and prognosis [6]. There is no established treatment for malignant PEComa as most regimens, based on chemotherapeutic responses of similar tumors such as clear cell sarcoma of soft parts (CCSSP) and GI stromal tumors (GIST), have largely been unsuccessful.

In this article, we present the first reported case of a PEComa of the upper extremity. We also report an encouraging response to doxorubicin-ifosfamide (Dox-Ifos), allowing for a more manageable definitive treatment with tumor excision.

## 2. CASE REPORT

**Figure 1:**
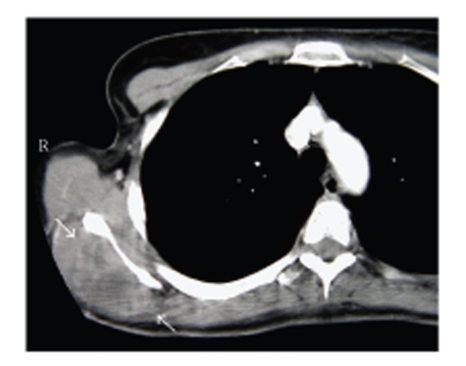
CT scan of the right scapula demonstrating a tumor in the infraspinatus fossa (arrows).

A 49-year-old female presented in April 2004 with a mass on the back of her right shoulder and complaints of worsening pain with activity and decreased ROM. The patient underwent MRI and CT imaging of her right scapula which revealed an abnormal 5.3 × 4.7 cm soft tissue mass in the posterior medial aspect of the infraspinatus muscle. (see Figure 1) The mass was hypointense on T1, hyperinterense on T2, and was associated with prominent surrounding edema and significant infiltration into the surrounding soft tissue. Biopsy of the mass in May 2004 revealed a high-grade malignant PEComa, largely undifferentiated and pleomorphic. Immunostaining of the tumor revealed strong positivity for Melan-A, no expression of S-100 or tyrosinase, and focal expression of HMB-45 and smooth muscle actin. The cells exhibited striking cytologic atypia and focal islands of necrosis, supporting the diagnosis of a high-grade malignant sarcoma. TNM staging determined by biopsy and imaging was T2bN0M0. Pathology consultation was sought from Dr. S. Weiss (Emory University), and yielded the same diagnosis.

**Figure 2:**
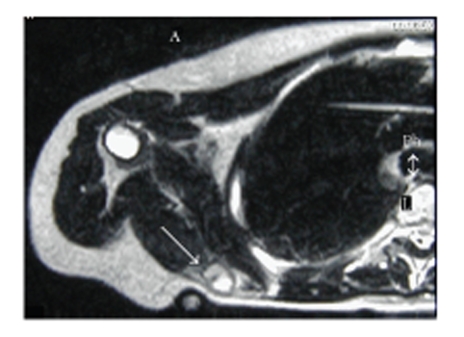
MRI of the right scapula showing a decrease in size of the PEComa after completion of neoadjuvant treatment (arrow). Edema of the surrounding infraspinatus muscle is evident as a bright area with less distinct boundaries than the tumor.

The patient began neoadjuvant therapy in May 2004, receiving six cycles of doxorubicin and ifosfamide (Dox-Ifos) between 5/10/04 and 9/10/04. Postchemotherapy MRI revealed an 80.1% decrease in size of the tumor to 3.3 cm × 1.5 cm. Subsequently, the patient underwent preoperative radiation therapy of the effected area consisting of five treatments per week for five weeks between 10/19/04 and 11/24/04 for a total of 5000 cGy. Postradiation MRI revealed an increase in the size of the mass of 45.5% to 4.0 cm × 1.8 cm (see Figure 2).

Limb sparing wide resection of the shoulder mass was performed on 12/20/04. Examination of the pathology specimen confirmed the diagnosis of malignant PEComa, with negative margins. The specimen revealed only 20% necrosis at the time of removal.

## 3. FOLLOWUP

On followup four months after the last chemotherapy treatment, the patient showed no evidence of disease on MRI. The patient's chief complaint of arm pain continued to improve. The patient exhibited no constitutional symptoms and was advised to continue to followup with repeat MRI of the scapula and Ct of the chest every three months for the first two years. The patient has no local recurrence thirteen months postresection (as of 1/06), but she did have a solitary lung mass on her chest CT at that same time. This lung mass excised fourteen months after removal of her primary lesion and was confirmed to be a metastasis.

## 4. DISCUSSION

PEComas are soft tissue tumors defined by their unique expression of both melanocytic and smooth muscle cell markers. Radial arrangement of epithelioid cells around a vascular lumen is characteristic in these tumors, although there can be variation in the relative proportion of epithelioid versus spindled cells. The group of tumors that share this “perivascular epithelioid cell (PEC)” nature is heterogeneous and includes the following: lymphangiomyomatosis, pulmonary and extrapulmonary clear cell sugar tumor, clear cell melanomyocytic tumor, and abdominopelvic sarcoma of the perivascular cells, perivascular epithelioid cell tumor (PECT) [9]. This tumor appears to have some predilection for female in their first-to-fourth decades. There has been a reported association with the tuberous sclerosis complex, but the vast majority of cases have been idiopathic. Because so little is known about the biology and natural history of this tumor, there is no established consensus regarding treatment. While most PEComa are benign, there have been several described cases of malignant or metastatic PEComa, most resulting in death after failing to respond to treatment. Features of malignancy include hypercellularity, high mitotic rate, presence of atypical mitotic figures, necrosis, and infiltrative growth pattern [9]. Rigby et al. recently described a failed attempt at treatment of a metastatic renal PEComa with DTIC and imatinib based on treatment of similar tumors and expression of c-kit [6]. Several cases of PEComa arising secondary to dedifferentiation of angiomyolipoma have been described, with variable response rates to doxorubicin and CyVADIC (cyclophosphamide, vincristine, epidoxorubicin, and dacarbazine) [10, 11]. For soft tissue sarcoma of the extremity, the mainstay of treatment continues to be surgical resection and radiation +/− chemotherapy. In this case, a regimen including all of these modalities was believed to offer the best chance for a disease-free outcome.

For our patient, doxorubicin, ifosfamide, and mensa were used as neoadjuvant therapy. These agents have been shown in numerous studies to be among the most efficacious in the treatment of adult soft tissue sarcoma [12–17]. Overall response rates to doxorubicin and ifosfamide have been reported as 21% and 28%, respectively [18]. The combination of the two drugs (Dox-Ifos) has been reported to be as high as 34–41% in various trials [19]. Here we present a case of a robust initial response by a high-grade malignant PEComa to this regimen, which to our knowledge, has not been described previously. Oddly enough, this response was not reflected in the posttreatment resection where there was only 20% necrosis. Despite this initial clinical response, our patient developed a single lung metastasis sixteen months after chemotherapy. Given the history of poor responses and outcomes of these tumors to other regimens, we believe that this case may represent a model by which future treatment may be designed.
